# Hydrophobic Amines and Their Guanidine Analogues Modulate Activation and Desensitization of ASIC3

**DOI:** 10.3390/ijms20071713

**Published:** 2019-04-06

**Authors:** Vasilii Y Shteinikov, Natalia N Potapieva, Valery E Gmiro, Denis B Tikhonov

**Affiliations:** 1 I.M. Sechenov Institute of Evolutionary Physiology and Biochemistry RAS, St. Petersburg 194223, Russia; potapieva2004@mail.ru (N.N.P.); denistikhonov2002@yahoo.com (D.B.T.); 2 Institute of Experimental Medicine, RAMS, St. Petersburg 197376, Russia; gmiro2119@gmail.com

**Keywords:** acid-sensing ion channel (ASIC), drug action, ligand-gated ion channel, pharmacology, small molecule, nociception, ASIC3

## Abstract

Acid-sensing ion channel 3 (ASIC3) is an important member of the acid-sensing ion channels family, which is widely expressed in the peripheral nervous system and contributes to pain sensation. ASICs are targeted by various drugs and toxins. However, mechanisms and structural determinants of ligands’ action on ASIC3 are not completely understood. In the present work we studied ASIC3 modulation by a series of “hydrophobic monoamines” and their guanidine analogs, which were previously characterized to affect other ASIC channels via multiple mechanisms. Electrophysiological analysis of action via whole-cell patch clamp method was performed using rat ASIC3 expressed in Chinese hamster ovary (CHO) cells. We found that the compounds studied inhibited ASIC3 activation by inducing acidic shift of proton sensitivity and slowed channel desensitization, which was accompanied by a decrease of the equilibrium desensitization level. The total effect of the drugs on the sustained ASIC3-mediated currents was the sum of these opposite effects. It is demonstrated that drugs’ action on activation and desensitization differed in their structural requirements, kinetics of action, and concentration and state dependencies. Taken together, these findings suggest that effects on activation and desensitization are independent and are likely mediated by drugs binding to distinct sites in ASIC3.

## 1. Introduction

Acid-sensing ion channels (ASICs) are cation channels from the degenerin/epithelial sodium channel (DEG/ENaC) superfamily. They are activated by fast acidification of the media, while prolonged exposure leads to their desensitization. There are five paralogous genes in this group, with the expression products of *ASIC1*, *2*, and *3* forming functional trimeric channels. *ASIC1* and *ASIC2* are predominantly expressed in the central nervous system, whereas *ASIC3* is more common in the peripheral nervous system [1]. The functions they fulfill also vary. ASIC1 and ASIC2 have been shown to contribute to the excitatory postsynaptic currents [2] and synaptic plasticity [3], and are also involved in the pathologic processes in stroke and ischemia [4,5]. On the other hand, ASIC3 is typically associated with peripheral nociception [6]. Another important difference is that ASIC3 channels, unlike other ASICs, do not fully desensitize during prolonged activation, supporting a significant sustained current [7]. 

The involvement of ASICs, in particular ASIC3, in the perception of pain has been firmly established in a number of studies (for review see [8]). The use of ASIC inhibitors in rats and humans was shown to alleviate cutaneous pain and hyperalgesia [9,10,11]. Surprisingly, knockout of the *ASIC3* gene in mice did not lead to a loss or significant decrease of their pain responses compared to wild type [12]. In fact, in the study of Kang et al. [13], triple knockout (for *ASIC1a*, *2* and *3* genes) mice showed enhanced pain sensitivity. This phenomenon can potentially be explained by the different roles of the ASIC channels in different species or particular levels and by specific details of their expression [14]. Large acidification-evoked currents were also shown in cardiac afferents, where they propagated cardiac pain and angina [15]. Characteristics of those currents are closely matched by heteromeric ASIC3/ASIC2b channels [16]. Other pain-associated conditions are also mediated by ASIC3, such as migraines [17], osteoarthritis [18], and muscle inflammation [19]. 

Given the importance of their role and the potential of new functions’ discovery, it is not surprising that ASIC pharmacology receives quite a lot of attention [20]. The pioneering paper by Waldmann et al. [21] described the action of amiloride, a common modulator of ENaC channels, which was found to be a low-affinity inhibitor of ASICs. Focusing on ASIC3 for the purposes of this work, there are several groups of drugs to be noted. The abovementioned amiloride inhibits peak currents of ASIC3 but does not affect the window current. Even more interestingly, in high concentrations it is capable of inducing said window current by itself, without acidification [22]. 2-Guanidine-4-methylquinazoline (GMQ) was also originally described as an ASIC3 modulator, although, unlike its predecessor, it has a potentiating effect. Like amiloride, GMQ can also evoke ASIC3 currents in neutral pH [23]. Later it was found that GMQ and its derivatives can also modulate ASIC1a [24]. 

Several endogenous compounds were shown to potentiate ASIC3 currents, including FMRFamides and related peptides [25], agmatine [26], and serotonin [27], with the last one only affecting the sustained component of the response. Agmatine was also able to activate the channels directly. 

On the other hand, toxins mostly display inhibitory action on ASIC3. A number of sea anemone toxins, such as APETx2 [28] and Ugr 9-1 [29], inhibit both peak and window currents in ASIC3. MitTx [30], which locks the channel in the open state, also works on ASIC3 but in significantly higher concentrations than on the other subunits.

In our research [31] we focused our attention on a group of small-molecule ligands we collectively called *hydrophobic monoamines*. Despite their structural simplicity, further investigations revealed quite complex effects that they can induce on ASIC channels [32]. We found that they can block the channel pore, affect the activation curve in either direction, and shift the desensitization curve to more acidic values, often with several effects observed for a single compound. Additionally, through this line of investigation a potential physiological modulator of ASICs (i.e., histamine) was discovered [33]. Its effects were specific to ASIC1a homomers. However, outside of initial assessment [31], the action of monoamines on ASIC3 was never studied. Thus, in the present work we attempted to elucidate the mechanisms of action of hydrophobic monoamines and their guanidine analogs on ASIC3 channels. Other compounds that were found to affect ASIC1a and/or ASIC2a, such as some antidepressants [34] and histamine receptor agonists [35], were also included in the study. 

## 2. Results

### 2.1. Drug Selection

Several groups of compounds were selected for the present study. The IEM line ofcompounds was originally designed as glutamate receptor agonists [36,37]. Their activity on ASIC channels was subsequently shown by our group [31,32]. Memantine [38] and 9-aminoacridine [39] also affect glutamate receptors as well as ASICs [31]. Other drugs included long-established antidepressants amitriptyline and tianeptine [40,41] and histamine receptor modulators imetit, dimaprit, and thioperamide [42,43]; their effects on ASICs were recently established in [34] and [35], respectively. It is important to note that in previous studies only the effects on ASIC1a and ASIC2a were examined, with ASIC3 covered very briefly in [31].

### 2.2. Estimation of Drug Activities

**Figure 1:**
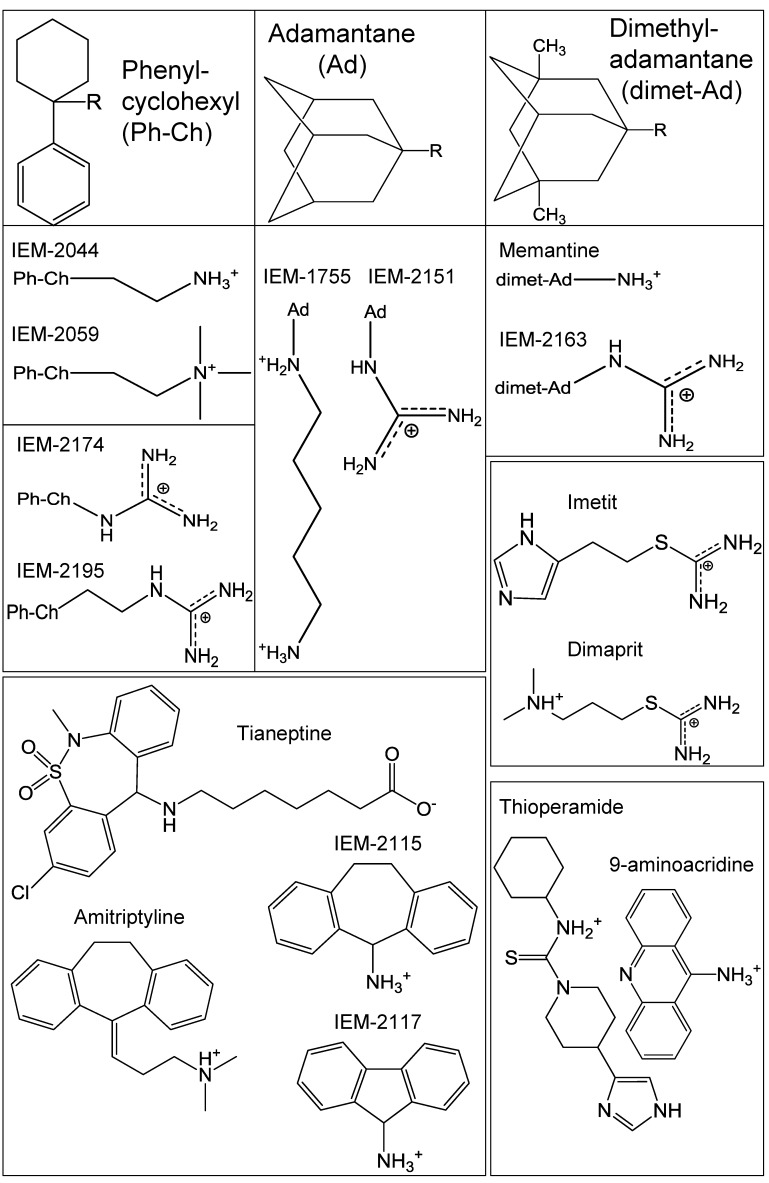
Chemical structure of the tested compounds. The first row represents common hydrophobic moieties (Ph-Ch, Ad, and dimet-Ad) of IEM compounds and memantine, with their terminal radicals (R) shown directly below.

For the sixteen compounds presented in Figure 1 we estimated the effects on peak and sustained currents evoked by acidification from pH 7.4 to 6.85, which caused 10% ± 7% (*n* = 11) of maximal peak response, and to pH 6.0, which caused 74% ± 16% (*n* = 11) maximal peak response. The compounds were applied simultaneously with acidification at a concentration of 0.5 mM. These applications were repeated 3–7 times to reach the effect’s equilibrium point and then 3–10 washout acidifications were done until complete recovery was achieved. 

**Figure 2:**
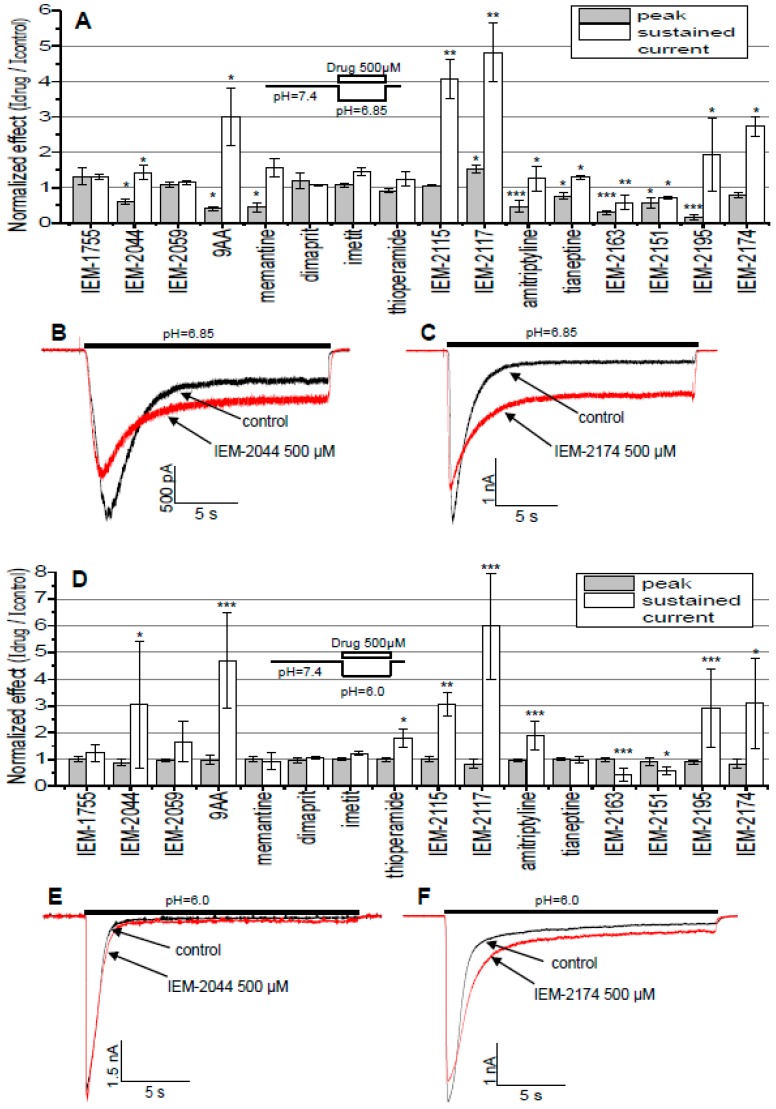
Estimation of the drug activities. Number of * denotes statistical significance at * *p* < 0.05, ** *p* < 0.01, or *** *p* < 0.001, respectively, *n* ≥ 5. (**A**) Effects of 500 µM of the compounds studied on the ASIC3 activated by pH drop from 7.4 to 6.85. Compounds were applied simultaneously with activation. (**B**,**C**) Representative examples of ASIC3 responses in control and in the presence of IEM-2044 (**B**) and IEM-2174 (**C**) when activated by pH drop from 7.4 to 6.85. (**D**) effects of 500 µM of the compounds studied on the ASIC3 activated by pH drop from 7.4 to 6.0. Compounds were applied simultaneously with activation. (**E**,**F**) Representative examples of ASIC3 responses in control and in the presence of IEM-2044 (**E**) and IEM-2174 (**F**) when activated by pH drop from 7.4 to 6.0.

The results are presented in Figure 2. At pH 6.85 (Figure 2A, with sample traces shown in Figure 2B,C) the peak component of the response was strongly inhibited by a number of compounds, the most potent being IEM-2195 at 85% ± 7% (*n* = 6) inhibition, and only IEM-2117 slightly potentiated the peak response by 42% ± 21% (*n* = 5). On the other hand, sustained current was typically potentiated, with the strongest effect by IEM-2117 at 382% ± 84% (*n* = 5). IEM-2163 and IEM-2151 were the only compounds that reduced the sustained current by 42% ± 21% (*n* = 9) and 29% ± 4% (*n* = 5), respectively. At pH 6.0 (Figure 2D, with sample traces shown in Figure 2E,F) the drugs’ effect on peak current disappeared, while for sustained current the overall picture stayed the same and the effects even somewhat increased in magnitude, with maximal potentiation by IEM-2117 reaching 498% ± 196% (*n* = 5) and inhibition by IEM-2163 at 54% ± 23% (*n* = 8). We can conclude from the data that (1) structural determinants of the effects on peak and sustained components of the response did not coincide and (2) only the effect on peak component demonstrated pronounced pH-dependence.

Typically, potentiation of the sustained current was accompanied by deceleration of the response decay, reflecting an effect on desensitization. In control experiments the decay time constant was 462 ± 143 ms (*n* = 8). The most drastic increase was seen for IEM-2195, which changed the decay time constant of the response to 4891 ± 1996 ms (*n* = 6). Inhibition of sustained current by IEM-2163 or IEM-2151 did not elicit significant changes of the response kinetics. 

### 2.3. pH and Concentration Dependencies

**Figure 3:**
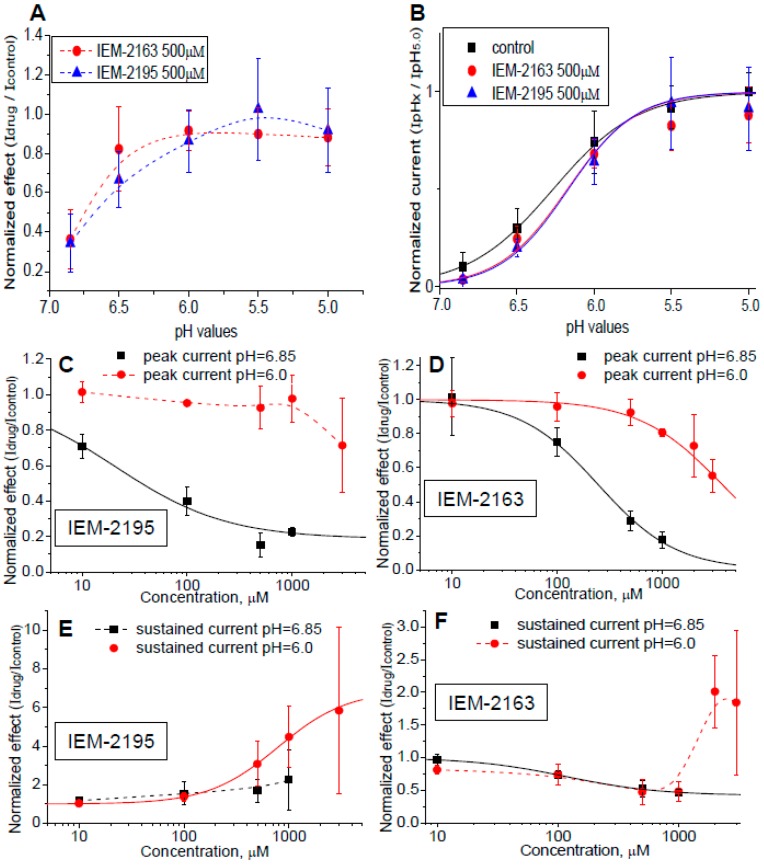
pH and concentration dependencies of IEM-2163 and IEM-2195 action. (**A**) Peak current inhibition was pH-dependent, the effects of both drugs disappeared under strong acidification. (**B**) IEM-2163 and IEM-2195 caused acidic shift of the ASIC3 activation curve. (**C**–**F**) Concentration dependencies of IEM-2163 (**D**,**F**) and IEM-2195 (**C**,**E**) action on peak (**C**,**D**) and sustained (**E**,**F**) currents. Fitting is shown in solid lines. Note that the concentration dependence of IEM-2163’s action on sustained current was biphasic at pH 6.0 where the current inhibition was small. Low concentrations caused inhibition, but at high concentrations the effect was inverted.

For detailed analysis we selected IEM-2163 and IEM-2195, as they demonstrate opposite effects (inhibition and potentiation, respectively) on the sustained currents. First, we estimated the pH-dependence of action on peak currents (Figure 3A). Figure 3B demonstrates that both compounds caused a parallel shift of activation to more acidic values without affecting maximal response. IEM-2163 at 0.5 mM shifted the pH_50_ value from 6.26 ± 0.02 in control to 6.17 ± 0.06. The shift caused by IEM-2195 was about equal, with the pH_50_ of activation being 6.17 ± 0.04 in the presence of this drug. 

We then studied the concentration dependencies of the actions of IEM-2163 and IEM-2195 on peak and sustained components of the response at pH 6.85 and 6.0 (Figure 3C–F). At pH 6.85, peak inhibition by IEM-2195 (Figure 3C)—which reflects its effect on activation—was well-fitted by the Hill equation, with optimal parameters nH = 0.82 ± 0.54, IC_50_ = 21 ± 15 μM. At pH 6.0, no significant effect was detected for concentrations up to 1 mM (Figure 3C). Our attempts to further increase the concentration led to poor clamp stability, resulting in highly diverging data at higher concentrations. In contrast, potentiation of the sustained current was well established at pH 6.0 (Figure 3E), where inhibition of activation was absent. The fitting resulted in EC_50_ = 784.2 ± 122.8 μM, nH = 1.31 ± 0.06, and maximal effect 588% ± 55% potentiation. 

IEM-2163 also strongly inhibited peak current. The IC_50_ at pH 6.85 (Figure 3D) was 245.64 ± 16.62 μM, nH = 1.17 ± 0.08. Similar to IEM-2195, peak inhibition at pH 6.0 was not significant. Sustained currents were significantly inhibited by IEM-2163 at pH 6.85 as well (Figure 3F), IC_50_ = 117.3 ± 5.6 μM, nH = 1.12 ± 0.07. A peculiar concentration dependence was observed for IEM-2163’s action at pH 6.0. Low concentrations caused progressive inhibition, but at around 1 mM the effect reached saturation at the level of 52% ± 16% of inhibition (*n* = 5), and at 3 mM, despite large data diversity, we saw an apparent potentiation by 84% ± 111% (*n* = 5). To ensure this was not an artifact of data variation, we performed additional experiments at 2 mM, which complied with the observed reversion of the effect resulting in 101% ± 56% (*n* = 6) potentiation.

An explanation of such concentration dependencies could be that they reflect a mixture of two distinct effects: pH-dependent inhibition of activation, which is responsible for the inhibition of peak component and window component in low concentrations; and reduction of desensitization, which determines the potentiation of the sustained current at high concentrations. Thus, analysis of concentration dependencies provided arguments in favor of the independence of drug effects on activation and desensitization.

### 2.4. Dependence of Action on the Application Protocols

**Figure 4:**
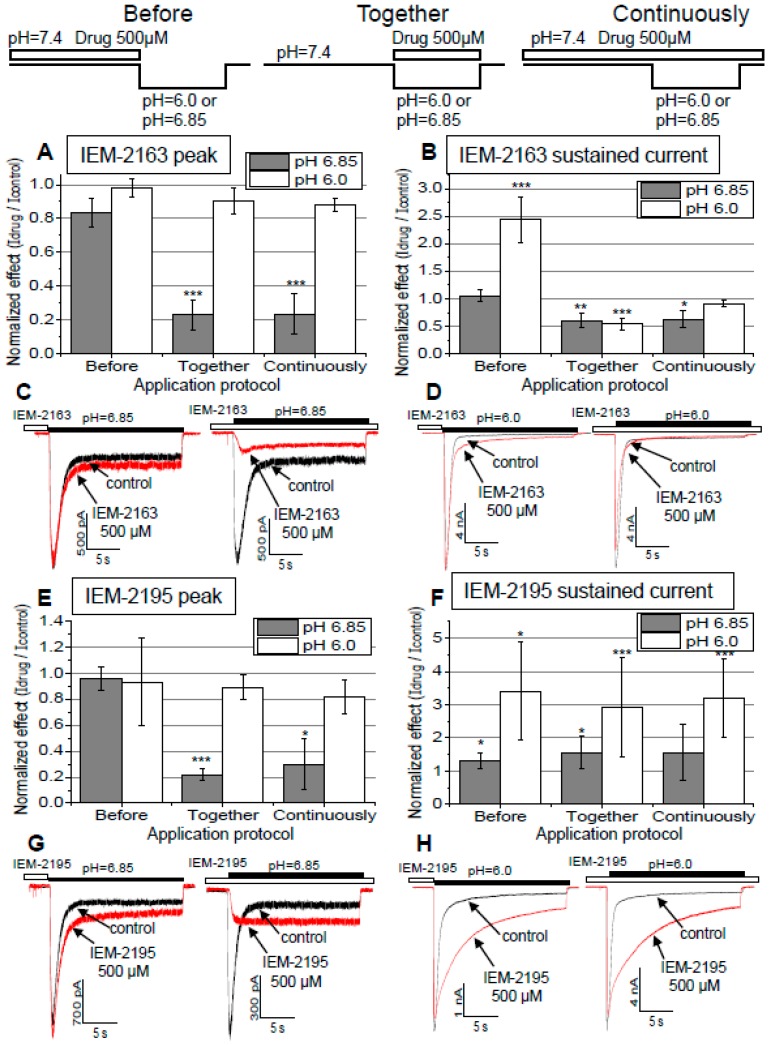
Effects of drug application protocol on peak and sustained currents of ASIC3 in different activating pH. Number of * denotes statistical significance at * *p* < 0.05, ** *p* < 0.01, or *** *p* < 0.001, respectively, *n* ≥ 5. (**A**) Effects of IEM-2163 on peak current and (**B)** sustained current. (**C**,**D**) Representative examples of ASIC3 responses in different activating pH (6.85 in (**C**) and 6.0 in (**D**)) and application protocols (left panels: application before activation, right panels: continuous application). (**E**–**H**) Same for IEM-2195. IEM-2163 (**A**–**D**) had mostly similar effects regardless of the protocol used, inhibiting peak current at pH 6.85 and sustained current for both pH values, with two notable exceptions. When applied before activation with activating pH 6.85 it had no effect at all, and in the same protocol but with activating pH 6.0 it strongly potentiated sustained current. IEM-2195 (**E**–**H**) mostly had a similar profile, but it typically potentiated the sustained current, although this effect was significantly weaker at pH 6.85, essentially disappearing when applied before activation.

Next, we compared drug effects in different application protocols. In addition to the protocol of simultaneous application (see above) we applied the drugs continuously or during 30 s immediately before activation by pH drop. The results are shown in Figure 4. The peak response evoked by pH 6.0 was not strongly affected, regardless of the application protocol for both compounds. More interestingly, peak response evoked by pH 6.85 was inhibited only if the drug was present during acidification and not only before it (Figure 4A,E). This finding can be explained by two different mechanisms: (1) the compounds interact only with the open channels and/or (2) the kinetics of their action is very fast. The effects on the sustained currents also depended on the application protocol. Application of IEM-2163 before activation by pH 6.0 resulted in 143% ± 41% (*n* = 5) potentiation, while under other conditions the drug caused inhibition (Figure 4B). For IEM-2195, in all protocols potentiation at pH 6.0 was higher than at pH 6.85 (two-way ANOVA for “protocol” and “pH” as factors, F(1,36) = 25.838, *p* < 0.001). This complex behavior is readily explained by the existence of two separate effects: pH-dependent inhibition of activation, which was also seen as peak inhibition; and pH-independent reduction of desensitization, with the total effect on the sustained currents being a sum of them.

As we diminished inhibition by the use of pH 6.0 for activation or by drug application only at neutral pH before activation, the anti-desensitizing effect on the sustained current increased (for IEM-2195) or became apparent (in the case of IEM-2163).

### 2.5. Kinetics of Action

**Figure 5:**
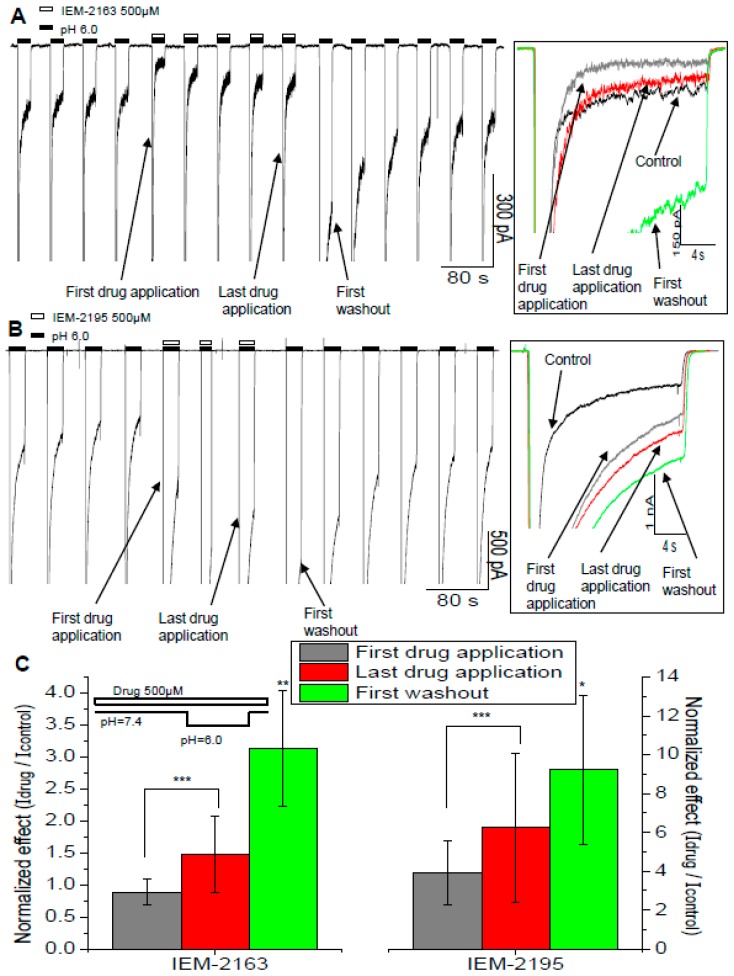
Non-monotonic effect development and washout of IEM-2163 and IEM-2195. (**A**,**B**) Representative recordings of the time course of experiments with IEM-2163 (**A**) and IEM-2195 (**B**). To the right are overlaid responses from the main panel. (**C**) The effect was the most pronounced in the protocol of continuous application. Number of * denotes statistical significance at * *p* < 0.05, ** *p* < 0.01, or *** *p* < 0.001, respectively, *n* ≥ 5. The average values were calculated as the ratio of amplitudes for the first drug application, last drug application, and first washout to the last control response, respectively.

Observation of the drug effects throughout the series of activations in the drug presence revealed an interesting tendency (Figure 5). Unlike the typical monotonic effect development, in experiments with IEM-2163, the sustained current was strongly inhibited during the first activation in the presence of the drug, but in subsequent activations the inhibition became less pronounced. The washout process was also non-monotonic—in the first activation it demonstrated a significant “overshoot”—the response decay was much slower than in control, resulting in the current at the end of activation being higher than the control one (Figure 5A). The control parameters were eventually reached after 5–7 activations. For IEM-2195, its potentiation developed monotonically but, similarly to IEM-2163, there was also a washout “overshoot”—in the first washout activation the current at the end of the response was even higher than in the last activation with the drug (Figure 5B). 

This phenomenon was the most pronounced with continuous drug application (Figure 5C). However, if the drugs were applied only before the channel activation, the overshoot effect disappeared. In this case both IEM-2163 and IEM-2195 caused potentiation, and recovery from it developed monotonically.

Our explanation of these effects is that inhibition of activation is fast while the effect on desensitization is much slower. Inhibition develops during the first activation in the presence of the drugs and is just as rapidly washed out during the first activation without them, while the reduction of desensitization requires several minutes to develop and wash out. This explanation agrees with the protocol dependence—fast peak inhibition required the drug’s presence during activation, whereas a slow effect on desensitization could be obtained during long pre-application and remained even if the drug was absent from the solution during the activation. Thus, the effects on activation and desensitization differed not only in structural determinants (Figure 2) as well as concentration and pH dependence (Figure 3), but also in their kinetics.

### 2.6. Biphasic Drug Effects, when Applied Exclusively to the Sustained Current

**Figure 6:**
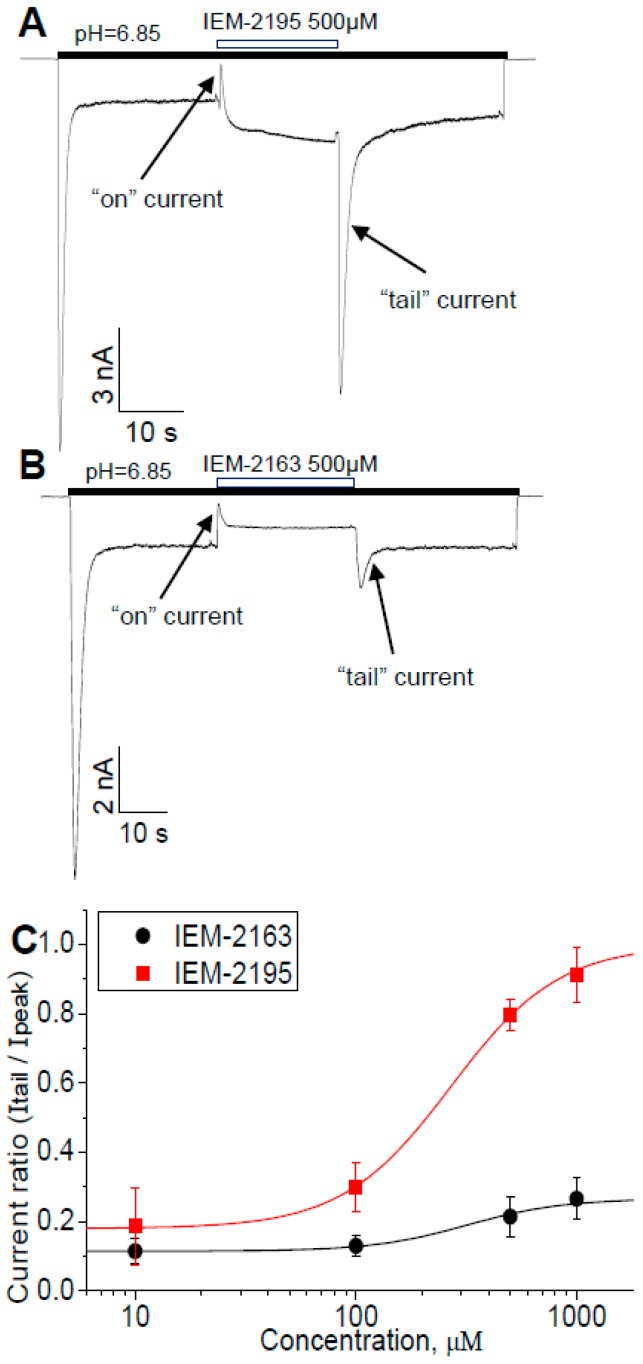
IEM-2163 and IEM-2195 cause transient currents when applied to sustained response. (**A**,**B**) Representative recordings. Fast drug application caused transient current decrease (“on” current), while washout induced transient increase (“tail” current). These transients reflect the presence of two opposite effects with different kinetics. (**C**) Concentration dependencies of “tail” currents.

The fact that ASIC3s do not desensitize completely and can mediate significant sustained current allows for one more type of experiment (Figure 6), which is helpful for the analysis of the mechanism of action. We activated the channels in the absence of a drug and only applied it when the current reached the sustained level. Washout was also performed during this prolonged activation, without returning to the neutral pH. Typical currents are presented in Figure 6A,B. Application of IEM-2195 (Figure 6A) caused fast transient “on current”, which then slowly returned to the equilibrium level similar to the value of sustained current potentiation observed in the previous experiments. Removal of the drug resulted in a large transient “tail current” before returning to the control value. Application and removal of IEM-2163 caused similar “on“ and “tail” transient currents, although they had smaller amplitude (Figure 6B). The main difference between the drugs was the direction of the change in the sustained current’s amplitude at the equilibrium level, which was potentiated by IEM-2195 and inhibited by IEM-2163, respectively. We were especially careful to ensure that this unusual behavior was not an artifact of the solution exchange. Additionally, “tail” currents for both compounds demonstrated clear concentration dependence (Figure 6C), with fitting resulting in EC_50_ = 269.06 ± 15.35 μM, nH = 1.76 ± 0.11 for IEM-2195 and EC_50_ = 319.69 ± 44.08 μM, nH = 2.00 ± 0.33 for IEM-2163.

We suggest that the observed “on” and “tail” currents reflect kinetics and complex mechanisms of drug action, which include the inhibition of activation and reduction of desensitization. We suggest that the “on” current appears because inhibitory action develops quickly, while slow modulation of desensitization is responsible for the subsequent equilibrium level of the sustained current. The change of this equilibrium effect (potentiation by IEM-2195 and inhibition by IEM-2163) may depend on the balance between these two opposite actions. Fast inhibition of activation would also be responsible for the “tail” currents, resulting from an acidic shift of activation (see Figure 3B). Purportedly, in this case the drug-bound channels would remain in the resting state even under conditions of acidic pH. Thus, fast removal of a drug would allow protons to bind and activate the channels. 

**Figure 7:**
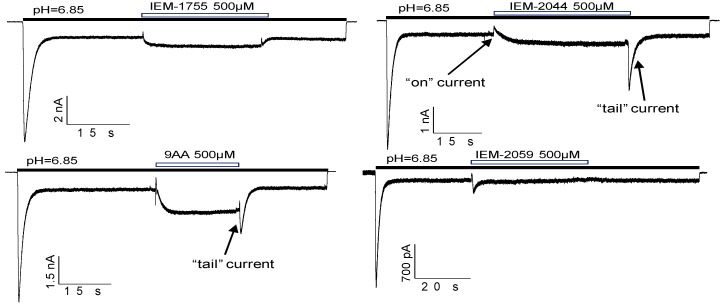
“On” and “tail” currents for different drugs. Transient currents were pronounced for the drugs which caused peak inhibition at pH 6.85 due to the activation shift (9AA, IEM-2044, see Figure 2A).

To further check this suggestion, we performed analogous experiments with some other drugs (Figure 7). 9-Aminoacridine and IEM-2044, which inhibit peak and potentiate the sustained component of the response, also demonstrated pronounced “on” and “tail” currents. In contrast, for IEM-2059 and IEM-1755 these transient currents were absent. In analogous experiments with agmatine performed by Li et al. [26], no “on” or “tail” currents were shown, probably because agmatine leads to an alkaline shift of activation and has an overall potentiating effect on ASIC3 currents.

## 3. Discussion

In the present work we demonstrated that many hydrophobic monoamines and their guanidine analogs affected ASIC3 in submillimolar concentrations. Two of them, IEM-2163 and IEM-2195, were studied in detail, whereby we found that their effects are best explained by the existence of two distinct mechanisms. The first mechanism is the acidic shift of activation that results in fast pH-dependent peak inhibition. The second one is deceleration of the ASIC3 desensitization, which raises the equilibrium level of the sustained current, thus effectively increasing its amplitude. The total drug effect on the sustained current depended on the ratio of these two independent types of actions.

We found that in a large series of drugs there was no correlation between these two types of action. Effect on activation was found to be pH-dependent, whereas modulation of desensitization was similar at pH 6.85 and pH 6.0. Elucidation of these types of action in turn required different application protocols. Concentration dependencies were also apparently separated, with effect on activation developing at lower concentrations than the effect on desensitization. There was also a drastic difference in kinetics, as the effect on activation was much faster than on desensitization. Taken together, these data suggest that two distinct types of action are mediated by drugs binding to different sites.

Drug effects on ASIC3 have been the subject matter of numerous studies. For instance, a detailed examination of GMQ and a representative series of its derivatives [44] allows for comparison with our data. In particular, compounds containing two aromatic rings and a guanidine group used at a concentration of 1 mM induced an acidic shift in the activation curve of ASIC3, similarly to IEM-2163, IEM-2195, and some other compounds in our work (Figure 2). On the other hand, GMQ and a few other derivatives induced an alkaline shift of activation, while we found no such effect for our compounds. Amiloride, a known ASIC blocker, also causes an alkaline shift of activation in high concentrations (0.5–1 mM). [24]. A similar but much weaker effect was induced by agmatine [26].

Notably, to detect a shift of activation it is necessarily to study the ligands’ effects with both weak and strong acidifications, and such data are not available for a number of other compounds.

Analysis of drugs’ action on sustained current is more complex, as it can be mediated by the effects on both channel activation and desensitization. Additionally, in some experimental setups ASIC3 does not mediate such currents in control, complicating quantitative estimations of effects. Various drugs, including GMQ [23], agmatine [26], and amiloride [22], induce or potentiate sustained ASIC3-mediated currents evoked by modest acidifications. Serotonin [27] and FMRFamide [25] potentiate the sustained current, while simultaneously slowing down desensitization kinetics under conditions of strong acidification. Note that these two compounds did not affect the peak component. In the present work we showed that drugs reduced the speed of response decay and increased final equilibrium level of sustained current amplitude under conditions of modest acidifications. We also experimentally separated this effect from their influence on activation, allowing us to detect such an effect for IEM-2163 despite its total inhibitory action. We are not aware of the proven examples of the compounds inhibiting the sustained current via modulation of desensitization. 

In this regard it is interesting to compare drugs’ effect on ASIC1a and ASIC3. In our previous paper [32] we demonstrated that many monoamines and their guanidine analogs affect the steady-state desensitization of ASIC1a by shifting its pH dependence to more acidic values, although this effect does not lead to the appearance of sustained current. The opposite effect, alkaline shift of the steady-state desensitization, was not revealed for small molecules but only for psalmotoxin [45]. Thus, if we assume that a similar process underlies desensitization in both ASIC1a and ASIC3, there is an apparent commonality in the direction of drug action, although it manifests differently, according to the channel type. In contrast, the drug action on activation properties is notably diverse. For instance, IEM-2044 and amitriptyline have opposite effects on different channels, inducing an alkaline shift of activation on ASIC1a [32,34] and an acidic one on ASIC3, while 9AA shifts the activation to more acidic values in both cases [32]. Histamine only enhanced the activation of ASIC1a and was inactive against ASIC3 [33]. Thus, we do not see a correlation for action on activation of ASIC1a and ASIC3. Similarly, in [44], GMQ and its derivatives also demonstrated varying effects on ASIC1a and ASIC3, with some compounds acting differently on different channels and others having the same effect regardless of the target.

The problem of the binding site(s) of ASIC ligands in the extracellular domain is intensely debated. According to recent structural data, channel “activation involves ‘closure’ of the thumb domain into the acidic pocket, expansion of the lower palm domain and an iris-like opening of the channel gate. The linkers between the upper and lower palm domains serve as a molecular ‘clutch’, and undergo a simple rearrangement to permit rapid desensitization” [46]. Another study [47] suggests that the protonable residues in the acidic pocket affect ASIC pH dependence, but in the palm domain they are responsible for the regulation of desensitization kinetics as well as prevention of the sustained currents. Thus, different regions participate in complex allosteric interactions which contribute to activation and desensitization, which in turn significantly complicates estimation of ligands’ binding site(s). In addition, particular mutations can unequally affect different modes of ligands’ action. One such example is Glu-79 in the palm domain of ASIC3 [48]. While it has been shown to be a crucial element for direct opening of the channel by GMQ, its mutation did not elicit any changes in GMQ’s effects on activation, but instead altered GMQ’s influence on the channel inactivation. The effect of mutations on ligands’ binding and action can also be either direct or allosteric. These data, together with the complex structure–activity relationships revealed in the present and other studies, raise the possibility that low-weight drugs can bind to more than one site in the extracellular domain of ASICs. For instance, binding to the acidic pocket could control effects on activation, whereas binding to the palm domain could be responsible for desensitization effects.

In our work we have focused our attention primarily on the low-to-moderate (pH 6.85–6.0) acidification range. While the more powerful acidification (pH < 5.0) that is frequently used in other studies can indeed occur [49] in vivo, it typically accompanies severe conditions such as tumors and open fractures. However, physiological processes and less-drastic pathologies usually stay in the less-acidic pH range [50,51,52,53]. Additionally, the research of Salinas et al. [54] indicates that ASIC3 activations by different levels of acidification are facilitated by distinct mechanisms. If one were to assume that those mechanisms in turn mediate specific physiological responses, then our work shows potential for the development of state-dependent drugs, which would affect only the specific response, without influencing other channel functions.

## 4. Materials and Methods 

### 4.1. Chemicals and Synthesis 

The synthesis of IEM compounds was performed at the Institute of Experimental Medicine, Saint-Petersburg, Russia as described in References [55,56,57,58]. The rest of the drugs were obtained from Tocris Bioscience and Sigma Aldrich. 

### 4.2. Cell Culture and Transfection

Chinese hamster ovary (CHO) cells, purchased from Evrogen company (Evrogen, Moscow, Russia), were cultured in a humidified atmosphere of 5% CO_2_ at 37 °C. Standard culture conditions were used for cell maintenance (Dulbecco’s modified Eagle’s medium (DMEM), 10% fetal bovine serum, 5% gentamicin). Transfection of plasmid encoding rat ASIC3 subunit was done using Lipofectamine 2000 (Invitrogen, Carlsbad, CA, USA) following the manufacturer′s protocol. We received expression vectors encoding rat ASIC3 as a gift from A. Staruschenko [59]. Those vectors were described in Reference [60]. Cells were transfected with 0.5 mg rASIC3 cDNA + 0.5 mg eGFP per 35-mm dish to achieve the expression of homomeric channels.

### 4.3. Drugs and Solutions

Pipette solution was prepared as 100 mM CsF, 40 mM CsCl, 5 mM NaCl, 0.5 mM CaCl_2_, 10 mM HEPES, and 5 mM EGTA, and its pH was adjusted to 7.35 with CsOH. For cells’ perfusion, an extracellular solution with 143 mM NaCl, 5 mM KCl, 2.5 mM CaCl_2_, 2 mM MgCl_2_, 18 mM d-glucose, 10 mM HEPES, and 10 mM MES was used, with its pH adjusted to 7.4. Drug-containing solutions were prepared from extracellular solution and their pH was adjusted again if necessary. 

### 4.4. Electrophysiology

Electrophysiological experiments were performed 48–72 h after transfection. Green fluorescence detected with a Leica DMIL microscope was used to identify transfected cells. Current recordings were acquired with an EPC-8 (HEKA Elektronik, Lambrecht, Germany) patch clamp amplifier in whole-cell voltage-clamp mode at a membrane potential of −80 mV. The data were stored on a personal computer via Patchmaster software (HEKA Elektronik, Lambrecht, Germany). The recordings where access resistance and capacitance changed by more than 10% over the course of the experiment were excluded from the analysis. 

### 4.5. Experimental Protocol

A standard experiment included a 30 s application of conditioning solution, followed by 20 s of activating solution. Then, the process was repeated until at least three responses in a row were differing from each other by less than 10% of their amplitude. In experiments where the drug was applied during conditioning period, we had to reduce its potential effect on the open channels. To achieve this, channel activation was preceded by a 3 s flush of drug-free conditioning solution.

When the drug was applied only to the sustained current, both activating solution and the drug were applied until sustained current stabilized, with no other time constraints. 

To account for response variability during the experiment, the control responses were averaged before drug application and after washout. 

### 4.6. Data Analysis and Statistics 

The values in the text are given as mean ± standard deviation (SD) with *n* ≥ 5. To test for effects’ significance, paired *t*-tests (drug versus control) or ANOVA were used, as appropriate, via the IBM SPSS Statistics software package (IBM, Armonk, NY, USA). OriginPro 8.1 (OriginLab Corporation, Northampton, MA, USA) was used for fitting of the data. 

## Abbreviations

ASIC	Acid-sensing ion channel
CHO	Chinese hamster ovary (cells)
DEG/ENaC	Degenerin/Epithelial sodium channels
GMQ	2-Guanidine-4-methylquinazoline
